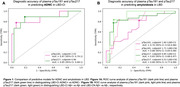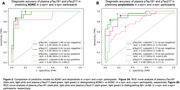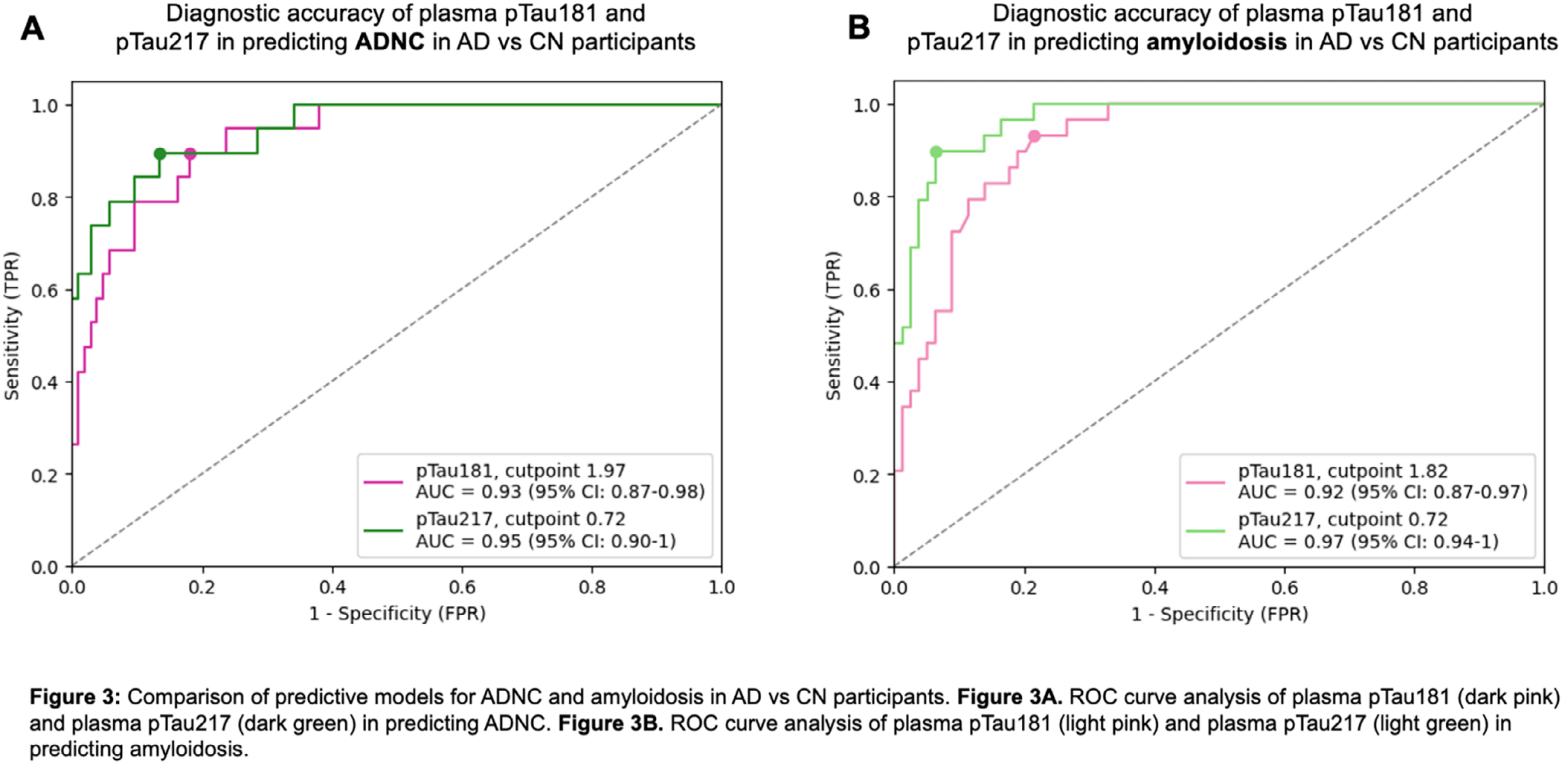# Comparing the diagnostic performance of plasma pTau181 and pTau217 for the identification of Alzheimer’s disease co‐pathology in Lewy Body Disease

**DOI:** 10.1002/alz.091458

**Published:** 2025-01-09

**Authors:** Alena Smith, Melani J. Plastini, Nicholas J. Ashton, Laia Montoliu‐Gaya, Edward N. Wilson, Burak Arslan, Christina B. Young, Joseph R. Winer, Marian Shahid, Hillary Vossler, Geoffrey A. Kerchner, Brenna Cholerton, Katrin I. Andreasson, Maya Yutsis, Victor W. Henderson, Thomas J. Montine, Lu Tian, Elizabeth Mormino, Henrik Zetterberg, Kathleen L. Poston, Carla Abdelnour

**Affiliations:** ^1^ Stanford University School of Medicine, Stanford, CA USA; ^2^ NIHR Biomedical Research Centre for Mental Health & Biomedical Research Unit for Dementia at South London & Maudsley NHS Foundation, London UK; ^3^ Wallenberg Centre for Molecular and Translational Medicine, University of Gothenburg, Gothenburg Sweden; ^4^ Department of Psychiatry and Neurochemistry, Institute of Neuroscience and Physiology, The Sahlgrenska Academy, University of Gothenburg, Mölndal, Gothenburg Sweden; ^5^ King’s College London, Institute of Psychiatry, Psychology & Neuroscience, Maurice Wohl Clinical Neuroscience Institute, London UK; ^6^ Stanford University, Stanford, CA USA; ^7^ Roche Pharma Research and Early Development, Roche Innovation Center, Basel Switzerland; ^8^ Stanford University, Department of Statistics, School of Humanities and Sciences, Palo Alto, CA USA; ^9^ Department of Neurodegenerative Disease and UK Dementia Research Institute, UCL Institute of Neurology, Queen Square, London UK; ^10^ UK Dementia Research Institute at UCL, London UK; ^11^ Department of Psychiatry and Neurochemistry, Institute of Neuroscience and Physiology, the Sahlgrenska Academy at the University of Gothenburg, Mölndal Sweden; ^12^ Department of Neurology and Neurological Sciences Stanford University School of Medicine, Stanford, CA USA

## Abstract

**Background:**

Lewy body disease (LBD) often co‐occurs with Alzheimer’s disease neuropathological change (ADNC), which can be detected using plasma pTau181 and pTau217. Few studies have investigated these biomarkers in LBD, nor have studies investigated plasma pTau217 in Parkinson’s disease (PD) cognitively normal patients (LBD‐CN), or in alpha‐synuclein positive (asyn‐positive) participants. Furthermore, uncertainties remain regarding LBD‐specific cut‐points for these biomarkers.

We aimed to determine whether there is a difference in the diagnostic performance of plasma pTau181 and pTau217 for detecting ADNC and amyloidosis in LBD. We also determine whether cut‐points for these biomarkers in LBD differ from those for AD. Finally, we conducted a sensitivity analysis in asyn‐positive participants.

**Method:**

We included 230 Stanford research participants: 110 cognitively normal (CN), 43 LBD‐CN, 41 LBD with cognitive impairment (LBD‐CI), and 36 AD. Plasma pTau181 was measured with the Lumipulse G platform, and pTau217 with the ALZpath pTau217 assay. A‐syn positivity was assessed in CSF with SYNTap®. Diagnostic accuracy of pTau181 and pTau271 in distinguishing ADNC (determined by CSF pTau181/Aβ42) and amyloidosis (determined by CSF Aβ42/Aβ40 or amyloid‐β PET) were evaluated with receiver‐operating characteristic (ROC) analyses. The Youden index was used to determine optimal cut‐points in distinguishing ADNC and amyloidosis, and the DeLong test to compare model performance.

**Result:**

In the LBD‐CI group, plasma pTau181 and pTau217 had similar diagnostic performance in distinguishing ADNC+ from ADNC‐ (Figure 1A). Similarly, in the LBD‐CN and LBD‐CI groups, both biomarkers had similar diagnostic performance in distinguishing Aβ+ from Aβ‐ participants (Figure 1B). However, in the sensitivity analysis, pTau217 outperformed pTau181 for detecting Aβ+ in asyn‐positive participants (AUC: 0.88, 95%‐CI: 0.77‐1 vs 0.77, 95%‐CI: 0.64‐0.90, p=0.045) (Figure 2B). Finally, plasma pTau181 and pTau217 cut‐points for detecting ADNC and amyloidosis in LBD differed from those for AD (Figures 1‐3).

**Conclusion:**

We present, for the first time, the diagnostic accuracy of plasma pTau217 in LBD‐CN and asyn‐positive participants. Our results indicate that plasma pTau181 and pTau217 reliably detect concomitant ADNC and amyloidosis in LBD. Particularly, plasma pTau217 appears more sensitive for detecting amyloidosis in asyn‐positive participants. Additionally, our findings underscore the importance of establishing LBD‐specific cut‐points for AD biomarkers.